# Physical Training Increases Erythroferrone Levels in Men

**DOI:** 10.3390/biology10111215

**Published:** 2021-11-21

**Authors:** Inga Dziembowska, Małgorzata Wójcik, Jakub Bukowski, Ewa Żekanowska

**Affiliations:** 1 Department of Pathophysiology, Faculty of Pharmacy, Collegium Medicum in Bydgoszcz, Nicolaus Copernicus University in Toruń, Curie-Skłodowskiej 9, 85-094 Bydgoszcz, Poland; j_bukowski@o2.pl (J.B.); zhemostazy@cm.umk.pl (E.Ż.); 2 Institute of Health Sciences, Hipolit Cegielski State University of Applied Sciences in Gniezno, Ks. Kard. Stefana Wyszyńskiego 38, 62-200 Gniezno, Poland; malgo_wojcik@interia.pl

**Keywords:** erythropoietin, erythroferrone, physical activity

## Abstract

**Simple Summary:**

Intense physical activity contributes to an increased consumption of oxygen transported by red blood cells. The red blood cells’ differentiation and proliferation process is mainly stimulated by erythropoietin (EPO) and erythroferrone (ERFE), which are novel markers of erythroid activity. The purpose of this study was to assess the level of concentration of these hormones in athletes’ blood. Seventy-three clinically healthy men took part in this study. Participants were divided into groups according to their physical activity, as assessed by the questionnaire survey. The first group included 39 athletes, the second group included 18 men with moderate physical activity, and the third—16 men with a sedentary lifestyle. Men with a high level of weekly physical activity had significantly different concentrations of ERFE and EPO than men with insufficient weekly physical activity. Higher endogenic ERFE and EPO levels are indicators of increased erythropoiesis in the period of intensified physical activity. The results obtained suggest the important role of endogenic EPO in the process of adaptation to intense physical activity.

**Abstract:**

Intense physical activity contributes to an increased demand for red blood cells, which transport oxygen to working muscles. The purpose of this study was to assess the concentration of erythroferrone (ERFE), the novel marker of erythroid activity in athletes, during the beginning of their training season. The study group consisted of 39 athletes aged 23.24 ± 3.77 years. The study was carried out during the athletes’ preparatory period of the training cycle. The control group consisted of 34 healthy men aged 22.33 ± 2.77 years. The erythropoietic activity was evaluated by determining athletes’ concentrations of erythropoietin (EPO) and erythroferrone (ERFE). The level of physical activity was assessed using the International Physical Activity Questionnaire (IPAQ). In the athletes’ group, we observed higher concentrations of EPO (Me = 12.65 mIU/mL) and ERFE (40.00 pg/mL) compared to the control group (EPO: Me = 5.74 mIU/ml, *p* = 0.001; ERFE: Me = 25.50 pg/mL, *p* = 0.0034). The average intensity of physical exercise significantly differentiated the participants as far as EPO and ERFE concentrations. These results suggest that intense physical activity, at least at the beginning of the training season, may stimulate EPO production, which increases ERFE release. This seems to be an adaptative mechanism that provides adequate iron for enhanced erythropoiesis.

## 1. Introduction

Research has shown that erythropoiesis responds to physical activity. Exercise-induced erythropoiesis is reflected in the elevation of reticulocytes (premature erythrocytes) following physical training [1]. However, the maturation of reticulocytes in bone marrow takes 1–2 days; therefore, it is an inaccurate marker for determining immediate erythropoietic stimulation [1].

Based on established knowledge of the regulation of erythropoiesis, the primary focus of attention has been directed toward tracing circulating EPO levels. EPO is a glycoprotein hormone produced in the kidneys that stimulates the proliferation, differentiation, and maturation of erythroid progenitor cells (EPCs) in the bone marrow [2]. Previous studies have indicated that physical training leads to a mild, but temporary, increase in EPO concentrations [3,4]. The synthesis and secretion of EPO is primarily the result of hypoxia, inflammation, and endocrine system stimulation [5]. Research suggests that exercise can be employed as a model of temporary immunosuppression, which occurs during physical stress such as hypoxia [6]. Acute bouts of physical exercise also regulate the immune response, i.e., by transiently redistributing immune cells to peripheral tissues, resulting in a heightened state of immunocompetence [7]. However, it has already been proven that regular physical exercise enhances the immune function response, reinforces the antioxidative capacity, and reduces oxidative stress [8,9]. Furthermore, exercise leads to an increased destruction of RBCs with exercise, leading to compensatory erythropoiesis [10].

Numerous studies involving adult athletes practising altitude training have shown that the concentration of EPO in the blood can increase after several hours, whereas its peak occurs between 1 and 3 days from the beginning of such a taining. In the following days of a continuous reduction in oxygen partial pressure, a gradual decrease in EPO concentration is observed [11].

In 2014, Kautz et al. described a protein derived from erythroid precursor cells—erythroferrone (ERFE) [12]. The authors indicated that increased erythropoietic activity results in ERFE secretion by erythroblasts [12,13]. ERFE suppresses hepatic synthesis of the master iron-regulatory hormone, hepcidin, leading to increased availability of body iron resources for erythropoiesis [14].

Based on previous research, it can be concluded that the EPO response observed after a period of intense physical exercise may stimulate ERFE synthesis. It may be worthwhile to determine whether the level of physical activity influences EPO and ERFE concentrations. This would be particularly relevant to propose guidelines for people at risk of insufficient erythropoiesis.

Thus, the aim of the current study was the assessment of erythropoietic activity based on the erythropoietin and erythroferrone levels among athletes as compared to sedentary men.

## 2. Materials and Methods

### 2.1. Participants

The study was carried out on a total of 73 clinically healthy men (average age 22.97 ± 3.51 years). All participants were informed about the purpose of the research and familiarised with the research procedure, to which they gave their informed written consent. All participants performed biochemical tests of parameters characterising erythropoiesis and completed the International Physical Activity Questionnaire (IPAQ) to determine their physical activity characteristics. The study group consisted of 39 men with an average age of 23.24 ± 3.77 years, who regularly practised sports at least four times a week for a minimum of 1.5 h for the previous three years. Of the 39 athletes, 23 (61%) practised martial arts, including mixed martial arts (MMA), boxing, kickboxing, judo, taekwondo, and karate. The remaining 16 athletes (39%) practised team sports: football and basketball. All athletes had valid sport medical examinations.

The control group consisted of 34 healthy men: non-athletes aged 22.33 ± 2.77 years. The subjects were recruited through posters and information leaflets placed in public places (universities, clinics, and board game clubs). According to IPAQ, the inclusion criteria involved no abnormalities in the basic peripheral blood counts and, for the control group only, an insufficient level of physical activity. The exclusion criteria were as follows: smoking, chronic diseases, cancer, and taking medications or iron supplements. The study protocol was approved by the Bioethics Committee of the Nicolaus Copernicus University in Toruń functioning at Collegium Medicum in Bydgoszcz (permit No. KB/247/2014) and was performed following the ethical standards set forth in the 1964 Declaration of Helsinki and its later amendments.

### 2.2. Physical Activity Assessment

An IPAQ questionnaire was used to assess physical activity. It expresses physical activity in Metabolic Equivalent of the Task (MET)× min/week units, which permits the easy classification of respondents into one of three categories of activity: insufficient, moderate, and high [15]. The median physical activity for each group is presented in Table 1. In the group of sedentary men, 16 (47%) showed insufficient physical activity, and 18 (53%) showed a moderate level. All athletes (*N* = 39, 100%) declared physical activity at a high level.

### 2.3. Blood Analysis

The test material was peripheral blood collected in the morning (7.00–9.00 a.m.) after a half-hour of rest via venipuncture from an arm. The athletes were asked not to perform any physical exercise on the day of the examination and the day before the examination to exclude the potential confounding effect of an acute bout of physical exercise on results.

Serum erythropoietin concentration was measured by an enzyme immunoassay (ELISA) using the EPO ELISA assay from Roche Diagnostics GmbH, Germany. The test is used to quantify erythropoietin in human serum and plasma. Serum erythroferrone concentration level was determined by enzyme immunoassay (ELISA) using the ELISA Kit for Erythroferrone (SEU-540Hu) from Cloud Clone, Houston, USA. The test is intended for the quantitative determination of erythroferrone concentration in human serum; however, it is not validated. To confirm that this assay detects the expected increase in ERFE, we compared the results of ERFE of blood donors (32 men, aged 21–56) before blood donation and a week after the donation, as the maximum ERFE peak is expected in 9 ± 4 days [16].

Furthermore, the following iron metabolism parameters were determined using the enzyme-linked immunosorbent method (ELISA): ferritin concentration (DRG Ferritin Kit reagent kit EIA-187) from DiaMetra, Spello, Perugia, Italy) soluble transferrin receptor concentration (Human sTfR ELISA from BioVendor Laboratory Medicine Inc. Brno, Czech Republic), and hepcidin concentration (ELISA Kit for Hepcidin, CEB-979Hu from Cloud Clone, Houston, TX, USA).

### 2.4. Statistical Analysis

Statistical analyses were performed using STATISTICA v. 13.1 (Statsoft, Cracov, Poland). The compliance of the distribution of individual features with the normal distribution was tested using the Shapiro–Wilk test. Linear variables were presented as median (Me), minimum (Min), and maximum (Max). The values of categorised variables are presented by quantity (N) and percentage values. To analyse differences in individual subgroups, the Mann–Whitney U test (for the comparison of 2 groups) and the Kruskal–Wallis rank test (for the comparison of groups) were used. A linearised non-linear regression model was performed to assess the relationship between EPO and ERFE in the studied groups. Differences with a *p*-value < 0.05 were considered statistically significant.

## 3. Results

The athletes showed higher ERFE and EPO concentrations in comparison to the non-athletes (Table 1).

Serum ERFE and EPO concentrations among highly physically active men were significantly higher than those declaring physical activity at an insufficient level. Furthermore, we observed that, although there were no between-group differences in hepcidin and ferritin levels, athletes presented lower serum transferrin receptor (sTfR) levels (Me 1.18 µg/mL) when compared to non-athletes (Me 1.83 µg/mL; *p* = 0.0001).

**Figure 1:**
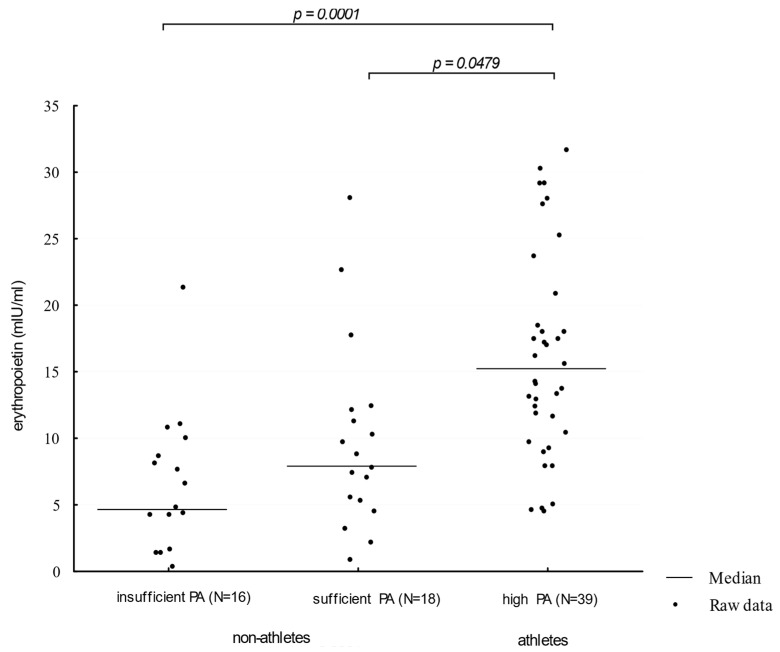
Erythropoietin levels in participants showing an insufficient, moderate, and high level of physical activity. PA—physical activity (η^2^ = 0.0811).

Athletes showed higher (Me = 16.32 mIU/ml) serum EPO concentration levels when compared to participants presenting moderate and insufficient physical activity (Me: 8.99 mIU/mL and 5.74 mIU/mL, respectively; *p* = 0.0001; η^2^ = 0.1521 and 0.2132, respectively). The comparison of EPO concentration distributions in groups of different physical activity levels is presented in Figure 1.

**Figure 2:**
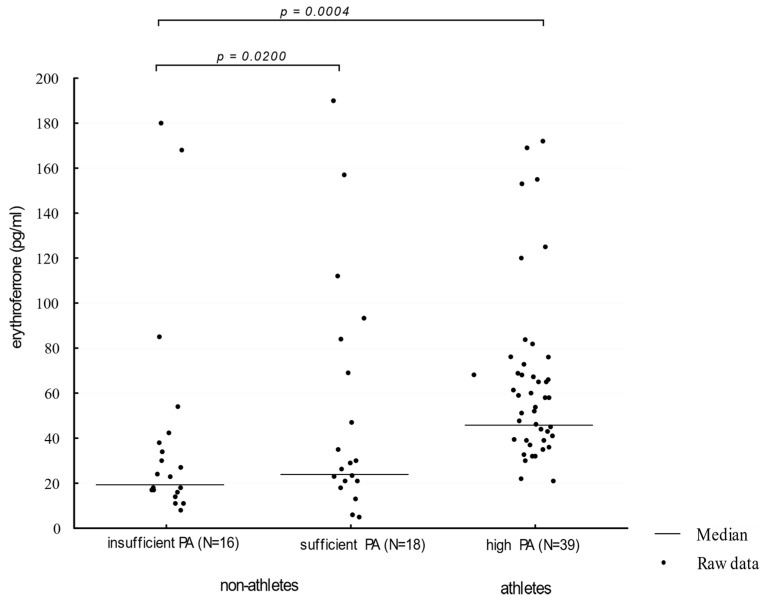
Erythroferrone levels in participants showing an insufficient, moderate, and high level of physical activity. PA—physical activity (η^2^ = 0.1230).

Athletes showed a higher (Me = 52 pg/mL) serum ERFE concentration when compared to participants presenting moderate and insufficient physical activity (Me: 30 pg/mL and 25.50 pg/mL, respectively, *p* = 0.0004; η^2^ = 0.0811 and 0.1232, respectively). The comparison of ERFE concentration distributions in groups of different physical activity levels is presented in Figure 2.

**Figure 3:**
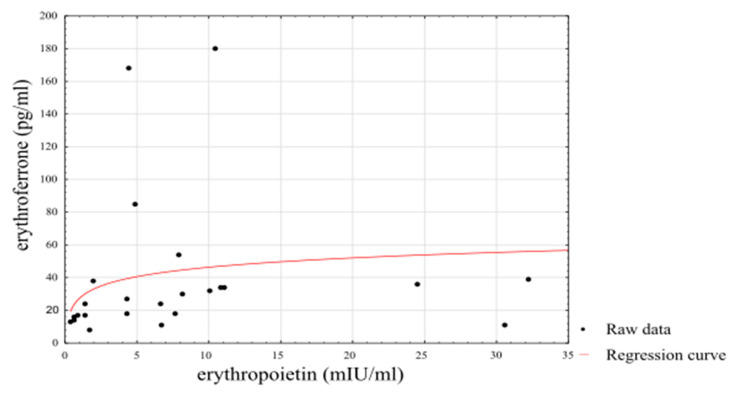
Linearised logarithmic regression analysis of ERFE concentrations in athletes.

Using a linearised logarithmic regression analysis, it was possible to set up a predictive model using an individual’s EPO level that explained 72% of the variance in ERFE levels among athletes (*β_stand_* = 0.85, *SE β_stand_* = 0.0691, *p* < 0.0001) and 39% of the variance in ERFE levels among non-athletes (*β_stand_* = 0.64, SE *β*_stand_ = 0.1625, *p* = 0.0006). The results of the logarithmic regression are presented in Figure 3.

## 4. Discussion

The aim of the current study was to compare EPO and ERFE levels between athletes and sedentary men. ERFE is produced by erythroblasts in response to EPO stimulation and mediates the inhibition of hepcidin synthesis [17]. A mouse model study showed that animals lacking the ERFE gene (*Erfe−/−*) developed anaemia; however, this phenomenon only affected the period of intensive growth [18]. Other studies in animal models have shown that bacterial pathogen-induced inflammatory anaemia, as well as increased erythropoiesis after significant blood loss, lead to increased ERFE synthesis [19]. Suppressing hepcidin expression with ERFE would exaggerate the availability of circulating iron for an increased iron demand for red blood cell haemoglobinisation [13]. In the present study, we observed greater ERFE levels among an examined group of athletes, which may suggest that intense physical effort following a recovery period enhances the erythropoietic activity, and thus ERFE synthesis. As the major factor determining ERFE production in erythroblasts is EPO, it is, therefore, possible that increased ERFE levels are connected with enhanced EPO synthesis in athletes. EPO is a hormone produced primarily in the kidneys and liver, and its most important role is to stimulate the proliferation, differentiation, and maturation of erythroid progenitor cells in the bone marrow [20]. During the last decade, researchers have also proven the local effects of EPO on blood vessel endothelium, nervous tissue, skeletal muscle, and heart muscle in response to physical or metabolic stress [21,22,23,24,25]. Based on these results, the role of EPO in the protection of nerve cells against oxidative stress during the process of neovascularisation is suggested [21,22,23]. EPO may also positively affect skeletal muscle regeneration, growth, and angiogenesis [21,22,23,24,25].

Expression of EPO mRNA is closely related to renal oxygen concentration, and hypoxia is considered the most important stimulus for EPO release [5]. Indeed, recent research showed that in elite athletes, EPO concentrations reached the highest value after 6 days of intermittent hypoxic exposure [24]. Previous studies also demonstrated the associations between muscle mass and erythropoiesis [25]. There are several lines of evidence supporting the concept that EPO augments exercise performance by activating an EPO receptor subtype in non-haematopoietic tissues, including skeletal muscle [26]. Recent research also draws attention to the small EPO fraction synthesised in skeletal muscle in response to intense physical exercise. Animal studies with mouse models have shown a 4–7 fold increase in EPO mRNA expression in skeletal muscle following exercise, and the increase was higher in glycolytic muscles and for trained mice [26]. The most recent data indicate that the endogenous EPO, through the EPO receptor in myocytes, controls mitochondrial biogenesis in skeletal muscle [27]. Nijholt et al. showed that a lack of EPO receptors in non-haematopoietic tissues lead to low mitochondrial content in skeletal muscles, as well as reduced myocyte growth and exercise capacity in response to voluntary exercise in mice [27]. Thus, it may be suggested that the source of erythropoietin released into circulation as a result of physical exertion may not only be from the kidneys but—at least in the early stages of exercise—from the working skeletal muscles as well [27].

Each bout of physical exercise contributes to the release of noradrenaline, cortisol, and androgens [28,29,30,31], which are hormones that stimulate the secretion of EPO, which, in turn, leads to the increased erythropoietic activity of the bone marrow [5]. Testosterone can also directly affect erythroblasts by increasing the number of receptors for EPO [28,29,30,31]. Current data have also revealed the complex interaction of noradrenaline within the bone marrow, and thus the dependence of the erythropoietic response on the intensity and duration of exercise and the associated stress response [28,29,30,31]. Review studies indicate that short-term exercise does not significantly affect EPO levels, while prolonged, intense physical effort (such as ultramarathon runs) leads to a temporary increase in EPO levels, both in trained and untrained men [4,31]. Therefore, a transient increase in EPO concentrations appears to have an adaptive significance to a substantial increase in physical effort compared to an individual’s usual physical activity.

Another potential cause of higher EPO levels could be increased hemolysis and/or iron deficiency as a result of increased physical activity. However, ferritin levels, which reflect the iron pool, were similar in both groups. Recent work focusing on the characteristics of iron metabolism proves that sTfR may be a more sensitive marker for assessing iron status in athletes than ferritin [32]. An increased demand for iron in the group of athletes would be reflected by higher sTfR values. Interestingly, the results of our analysis showed that the concentration of sTfR in athletes was significantly lower than in the group of non-athletes, which reflect even lower iron demand. To complete the analysis of the endocrine system that regulates iron homeostasis, we analysed the serum hepcidin concertation in both groups. A review of 21 studies that analysed hepcidin concentration in response to endurance exercise (running, cycling, rowing, and walking) showed that post-workout increases in hepcidin concentrations typically peaked between 3 and 6 h after training and lasted up to 24 h [33,34]. In the presented study, athletes were asked not to engage in physical training for about 24 h before blood collection. Therefore, it seems that the increase in hepcidin concentrations caused by physical exertion is a temporary state, and surprisingly, ERFE may play a role in returning hepcidin to normal values.

It is worth noting that the current study was conducted during the preparatory period of the training cycle, which is characterised by intensified physical activity following the recovery period. Thus, increased physical activity may stimulate erythropoiesis and greater ERFE and EPO levels in athletes in comparison to sedentary and moderately active men. This is in line with a previous review showing a significant variation in reticulocyte counts, which represents erythropoietic activity in athletes throughout the year [1]. The authors reported generally higher reticulocyte counts at the beginning of the season but lower values after intensive training sessions, competitions, and at the end of the season [1]. The elevated EPO levels in athletes observed in the current research reflect a temporary state associated with the adaptation of the musculoskeletal system to the change in the intensity and type of physical training. Mathematical models created in this work showed that EPO concentration could be used to predict ERFE concentration within the group of athletes, explaining more variance (R^2^ = 0.72) than within the group of sedentary men (R^2^ = 0.39). These results may indicate the role of ERFE as a protein that provides optimal iron reserves during exercise-induced, enhanced erythropoiesis in athletes.

Despite the undoubted value in using ERFE to attempt to explain the physiological mechanism of exercise-induced erythropoiesis, there were limitations of this study introduced by the homogeneity of the study groups with respect to age, sex, and eating habits. In addition, the analysed group of athletes is not representative of all physically active men or the broadly understood population of athletes, as it was limited to only a few sport disciplines. Furthermore, the athletes are not only “highly active” people, but they are also competitors and have some genetic peculiarities. In addition, we observed increased EPO and ERFE levels in athletes compared to non-athletic individuals at one time-point, whereas it would be interesting to investigate the time-course changes in these parameters over the entire training cycle.

## 5. Conclusions

Based on the conducted analyses and the explained directional variability, one can assume the possible role of erythropoietin in the activation of erythroferrone that is in line with previous literature reports. Higher concentrations of EPO and ERFE observed in athletes indicate that regular, intense physical activity may stimulate the erythropoiesis process.

## Figures and Tables

**Table 1:** Group characteristics and biochemical parameters for the test groups.

Variable	Athletes	Non-Athletes	*p*	η^2^
	N = 39	N = 34		
	Me (Min–Max)	Me (Min–Max)		
Age (years)	21.00 (18.00–32.00)	21.50 (18.00–29.00)	0.5321	0.0031
BMI (kg/m^2^)	23.12 (17.28–27.15)	23.61 (17.94–28.12)	0.2431	0.0000
Physical activity (MET × min/week)	5120.00 (3100.00–6300.00)	1230.00 (340.00–2100.00)	<0.0001	0.5706
Years of training	9.00 (4.00–14.00)	N/A	N/A	N/A
Serum EPO levels (mIU/mL)	12.35 (0.90–32.25)	5.68 (0.38–32.15)	0.0001	0.1612
Serum ERFE levels (pg/mL)	40.00 (5.00–190.00)	25.5 (8.00–180.00)	0.0042	0.1861
Serum hepcidin levels (pg/mL)	8.43 (5.81–12.54)	8.21 (5.92–12.32)	0.5431	0.0001
Serum ferritin levels (ng/mL)	57.36 (20.58–158.32)	45.28 (21.52–162.45)	0.3412	0.0013
Serum sTfR levels (µg/mL)	1.28 (0.81–3.43)	1.83 (1.47–2.78)	0.0002	0.1014

EPO—erythropoietin; ERFE—erythroferrone; sTfR—serum transferrin receptor.

## Data Availability

The data presented in this study are available on request from the corresponding author.
